# Protective Effects of Chronic Intermittent Hypobaric Hypoxia Pretreatment against Aplastic Anemia through Improving the Adhesiveness and Stress of Mesenchymal Stem Cells in Rats

**DOI:** 10.1155/2017/5706193

**Published:** 2017-07-16

**Authors:** Jing Yang, Li Zhang, Handong Wang, Zan Guo, Yixian Liu, Yi Zhang, Chuan Wang, Quanhai Li

**Affiliations:** ^1^Department of Physiology, Hebei Medical University, Shijiazhuang, Hebei 050017, China; ^2^Hebei Collaborative Innovation Center for Cardio-cerebrovascular Disease, Shijiazhuang, Hebei 050017, China; ^3^Department of Cardiology, Bethune International Peace Hospital, Shijiazhuang, Hebei 050017, China; ^4^Undergraduate of College of Basic Medicine, Hebei Medical University, Shijiazhuang, Hebei 050017, China; ^5^Department of Pharmacology, Hebei Medical University, Shijiazhuang, Hebei 050017, China; ^6^Cell Therapy Laboratory, The First Hospital of Hebei Medical University, Shijiazhuang, Hebei 050000, China; ^7^Department of Immunology, Basic Medical College, Hebei Medical University, Shijiazhuang, Hebei 050017, China

## Abstract

Aplastic anemia (AA) is a common malignant blood disease, and chronic intermittent hypobaric hypoxia (CIHH) has a beneficial effect against different diseases. The aim of the present study was to investigate the protective effect of CIHH against AA and underlying mechanisms. 5-Fluorouracil and busulfan treatment induced AA model in rats with reduction of hematological parameters and bone marrow tissue injury and decrease of the colony numbers of progenitor cells. CIHH pretreatment significantly reduced the incidence rate of AA and alleviated above symptoms in AA model. The adhesive molecules of bone marrow mesenchymal stem cells (BMMSCs) in AA model, VLA-4, VCAM-1, and ICAM-1 were upregulated, and those of CD162 and CD164 were downregulated by CIHH pretreatment. The expressions of HIF-1*α* and NF-*κ*B in BMMSCs were also decreased through CIHH pretreatment. Overall, the results demonstrated for the first time that CIHH has an anti-AA effect through improving the adhesiveness and stress of mesenchymal stem cells in rats. CIHH could be a promising and effective therapy for AA.

## 1. Introduction

Aplastic anemia (AA), an acquired bone marrow failure syndrome [1], manifests itself not only in excessive reduction of hematopoietic stem cells (HSCs) [2] and immune disorders [3, 4] but also in a deficiency of hematopoietic microenvironment (HIM) [5, 6]. Currently, there is growing interest in the adhesiveness of stem cells as a pivot for regeneration therapy against aplastic anemia.

The normal hematopoietic function of bone marrow needs both normal HSCs and normal hematopoietic microenvironment (HIM). The advanced research showed that improving HIM of bone marrow in AA patients will promote the effect of therapies for AA with a doubled result. Bone marrow stromal tissue is an important part of HIM, and bone marrow mesenchymal stem cells (BMMSCs) are the most important component cells of bone marrow stromal tissue, known as bone marrow stromal cells in the early literature. BMMSCs can secrete various hematopoietic factors supporting the proliferation and multidifferentiation in host HSCs [6]. So BMMSCs play an important nutritional support role on bone marrow HSCs and form the structural basis for the survival and executive function of bone marrow HSCs. In the process of bone marrow hematopoiesis, cell adhesion molecules (CAMs) mediate the combination between hematopoietic stem cells and bone marrow stromal cells or matrix molecules, which also can mediate the homing of hematopoietic stem cell [7]. Therefore, the therapy via enhancing the adhesiveness and nutritional support between the bone marrow hematopoietic stem cells and mesenchymal stem cells might be valuable for AA treatment.

Hypoxia is well known to promote the production of red blood cells [8]. Researches in vitro showed that hypoxia could promote the proliferation of HSCs and bone marrow mesenchymal stem cells (BMMSCs) and could inhibit their differentiation [9]. Hypoxia has been proved to mobilize multipotential mesenchymal stem cells into peripheral blood [10, 11]. Chronic intermittent hypobaric hypoxia (CIHH), a special kind of hypoxia simulating high-altitude hypoxia, has been demonstrated to have beneficial effects on the body. For example, CIHH protects the heart, the central nervous system, and the liver against ischemia/reperfusion or hypoxia/reoxygenation injuries [12–14]. Our previous studies showed that CIHH had no effects on basic cardiac function but protected the heart against ischemia/reperfusion injury [15–17]. Recently, our research showed that CIHH treatment had preventive and therapeutic effects on some diseases, such as collagen-induced arthritis [18, 19], renal vascular hypertension [20], and fructose-fed metabolic syndrome in rats [21], and on diabetes through improving liver insulin resistance in diabetic rats [22, 23]. However, the effects of CIHH on AA and their underlying mechanisms have yet to be elucidated.

Our present work is focused on the preventive effect of CIHH pretreatment on AA induced by the combination of 5-FU and BU in rats. The experimental hematology, the primary progenitor cell culture, the long-term bone marrow explant culture (LTBMC-Ex) methods, flow cytometric (FCM) analysis, and Western blotting method were used to investigate the protective effect of CIHH against AA and underlying mechanisms.

## 2. Materials and Methods

### 2.1. Preparation and Evaluation on AA Rat Model

Briefly, adult male Sprague-Dawley rats were given a 5-fluorouracil (5-FU) (J&K Scientific LLC, USA) injection (150 mg/kg) intraperitoneally in the first day. Then, busulfan (BU) (20 mg/kg) (Sigma-Aldrich, USA) was given intraperitoneally in the seventh day and was injected weekly. Fifty milligrams pure BU was dissolved in 5 mL acetone and was further diluted with 20 mL bacteriostatic water to make final concentration (2 mg/mL). The whole AA induction lasts 28 days. The values of red blood cell (RBC), white blood cell (WBC), and platelet (PLA) in peripheral blood should be decreased more than half at least. The evaluation of AA was mainly according to the examination of bone marrow, especially bone marrow biopsy [24–26].

### 2.2. Animal Grouping, CIHH Treatment, and Protocols

Adult male Sprague-Dawley rats were provided by the Hebei Key Laboratory of Laboratory Animal Science (Shijiazhuang, China). All animals were treated in accordance with the Guide for Care and Use of Laboratory Animals published by the US National Institutes of Health (NIH Publication number 85-23, revised 1996). All experimental procedures were reviewed and approved by the Ethics Committee for the Use of Experimental Animals at Hebei Medical University.

The adult male Sprague-Dawley rats (6 weeks, 180~220 g body wt, clean grade) were randomly divided into four groups: control (Con), CIHH pretreatment (CIHH), aplastic anemia induction (AA), and CIHH plus AA (CIHH + AA) groups. In AA rats (*n* = 20), aplastic anemia was induced by an injection of 5-FU and BU. CIHH rats (*n* = 20) were only treated with CIHH (simulated 3000 m altitude, 5 hours per day for 28 days, PO_2_ = 108.8 mmHg, from 8 : 00 to 14 : 00) [16]. The CIHH + AA rats (*n* = 20) were treated with CIHH before AA induction. Control rats (*n* = 20) received neither AA induction nor CIHH. During the experiment, the health condition and physical activity of the rats were monitored regularly. All rats were sacrificed with an overdose of pentobarbital sodium (100 mg/kg, i.v.) at 56d.

The whole duration for animal treatment lasts 56 days. For AA rats, coinjection of 5-FU and BU was given at 29d, and the samples of blood, bone marrow, and femora tissue were collected at 56d. For CIHH + AA rats, CIHH treatment was given from the 1st day to 28d, a 5-FU and BU coinjection was given at 29d, and the samples were collected to assess the outcomes at 56d. For CIHH rats, CIHH treatment was only given from the 1st day to 28d and then lived in normoxic condition until sacrificed at 56d. For control rats, a physiological saline injection was given at 29d and sacrificed at 56d in normoxic condition. During the experiment, the health condition and physical activity of the rats were monitored regularly. All rats were sacrificed with an overdose of pentobarbital sodium (100 mg/kg, i.v.) at 56d.

### 2.3. Analysis of Peripheral Hemogram in General

The blood sample was collected once a week until being sacrificed. Approximately 500 *μ*L of blood were collected by postocular venous plexus from each group using 500 *μ*L ethylene diamine tetraacetie acid (EDTA) anticoagulated. The values of RBC, WBC, PLA, HGB, and HCT were determined by using standard laboratory techniques.

### 2.4. Bone Marrow Histomorphology and Pathology

After adherent soft tissue and epiphyses were removed from the long bones (femur and tibia) of rats, the bone tissues from femur were fixed in 4% paraformaldehyde solution for 48 hours. Then, the samples of bone were immersed in 20% EDTA solution for 4 weeks for decalcification. After routine dehydration and paraffin embedding, the tissue was cut into 5 *μ*m thick sections that pasted on a polyuridylic acid-treated microscopic slide for 48 hours baking at 60°C and H-E staining. After H-E staining, hyperplasia of bone marrow hematopoietic tissue was observed under an upright microscope.

### 2.5. Culturation of Hematopoietic Progenitor Cells (HPCs)

BMMNCs were assayed for colony-forming unit-granulocyte-macrophage (CFU-GM), colony-forming unit-erythroid (CFU-E), burst-forming unit-erythroid (BFU-E), and colony-forming unit-mixed lineage (CFU-Mix) in semisolid culture medium. Briefly, BMMNCs were cultured in Iscove's modified Dulbecco's medium (Invitrogen, USA) containing 1% (wt/vol) methylcellulose (Sigma-Aldrich, USA), 1% (wt/vol) bovine serum albumin (BSA) (Sigma-Aldrich, USA), 1 × 10^−4^ M *β*-mercaptoethanol (*β*-ME) (Sigma-Aldrich, USA), 20% (vol/vol) screened fetal bovine serum (FBS) (Sigma-Aldrich, USA), and 30% (vol/vol) horse serum (HS) (Sigma-Aldrich, USA). The culture system was mixed and seeded into 24-well culture plates. Each sample was seeded into 3 wells. Each well contained 2 × 10^5^ BMMNCs with 0.1 *μ*g/mL rat granulocyte macrophage colony-stimulating factor (GM-CSF) (Sigma-Aldrich, USA), 5 IU/mL rat stem cell factor (SCF) (Sigma-Aldrich, USA), 1 IU/mL rat interleukin-3 (IL-3) (Sigma-Aldrich, USA), and 10 U/mL rat erythropoietin (EPO) (Sigma-Aldrich, USA). The total volume of culture system was 0.3 mL. Cells were incubated at 37°C in a humidified 5% CO_2_ atmosphere, and colonies were scored on day 3 for CFU-E (aggregates ≥ 50 hemoglobinized cells) [27], on day 7 for BFU-E (aggregates ≥ 200 hemoglobinized cells) [28], on day 10 for CFU-GM (aggregates ≥ 50 nonhemo-globinized cells) [29], and on day 12 for CFU-Mix (aggregates ≥ 50 hemoglobinized and nonhemoglobinized cells) [30].

### 2.6. Long-Term Bone Marrow Explant Cultures (LTBMC-Ex) In Vitro for Mesenchymal Progenitor Cells (MPCs)

Under sterile condition, small 0.2mm^3^ fragments of bone marrow were explanted to 75 mm culture dish containing 4 mL of RPMI-1640 supplemented with 30% FBS. The culture medium also contains 1% bovine serum albumin (BSA), 10^−4^ M *β*-mercaptoethanol (*β*-ME), 100 ng/mL rat stem cell factor (SCF), 50 ng/mL rat interleukin-3 (IL-3), and 50 ng/mL rat granulocyte macrophage colony-stimulating factor (GM-CSF) [31]. The cultures were incubated at 37°C in an atmosphere of 5% CO_2_ air for 4 weeks. After days 14, 19, and 25 of culture, the numbers of colonies deriving from CFU-Fs (aggregates ≥ 8 to 20 spindle-shaped nonhemoglobinized cells) [10, 31] were scored using an inverted microscope.

### 2.7. Generation of BMMSCs

BMMSCs were obtained from rat femoral BM as previously described [10]. Briefly, bones were cleaned of adherent soft tissue, epiphyses were removed, and marrow was harvested by inserting an 18-gauge syringe needle into one end of the bone shaft and seeded at a density of 2 × 10^6^ cells per mL in 10 mL Dulbecco's modification of Eagle's medium (DMEM) (Invitrogen, USA) culture medium 20% (vol/vol) screened fetal bovine serum (FBS). Cells were incubated at 37°C in a humidified 5% CO_2_ atmosphere. All nonadherent cells were removed by changing the medium during 48 hours; thereafter, medium was changed twice a week. The monolayer of adherent cells was trypsinized by 0.25% trypsin-EDTA when it was half confluent, resuspended in culture medium, and seeded at 1 × 10^4^ cells per mL in 10 mL DMEM culture medium at 37°C in a humidified 5% CO_2_ atmosphere again (passage 1 [P1]).

### 2.8. Flow Cytometric Analysis of the Surface Markers on BMMSCs

Flow cytometric analysis was performed to evaluate the surface markers of BMMSCs with a FACSCalibur flow cytometer (FACS Canto™ II, BD Biosciences, USA) using a 488 nm argon laser. Cells from single-cell suspension were incubated for 60 minutes at 4°C with monoclonal antibodies (Abs) against rat antigens, including CD90, CD44, CD73, CD34, and CD45. Irrelevant isotype-identical Abs (clone F8–11-13; Serotec) served as negative control. Specific and unspecific Ab binding was detected with a secondary phycoerythrin-labeled anti-mouse Ab (Serotec). Samples were analyzed by collecting 10,000 events using Cell-Quest software (Becton, Dickinson and Company).

### 2.9. Measurement of the Expression of Very Late Antigen-4 (VLA-4), Vascular Cell Adhesion Molecule-1 (VCAM-1), Intercellular Cell Adhesion Molecule-1 (ICAM-1), CD162, and CD164 in BMMSCs

The protein expression of VLA-4, VCAM-1, ICAM-1, CD162, and CD164 on passage 2 (P2) BMMSCs was analyzed by FCM and Western blotting. Firstly, the changes in expression of adhesion molecules were determined by fluorescent intensity using FCM. The BMMSC suspensions are divided into 6 tubes and the corresponding fluorescence-labeled monoclonal antibodies (5 *μ*L): FITC anti-rat VLA-4 monoclonal antibody (eBioscience, USA), FITC anti-rat VCAM-1 monoclonal antibody (eBioscience, USA), FITC anti-rat ICAM-1 monoclonal antibody (eBioscience, USA), FITC anti-rat CD162 monoclonal antibody (eBioscience, USA), FITC anti-rat C164 monoclonal antibody (eBioscience, USA), and IgG (eBioscience, USA) were added into the tubes containing 50 *μ*L BMMSC suspensions, respectively. There were about 5 × 10^5^ cells in each tube. The contents in the tubes were mixed vertically and kept for 30 minutes at room temperature in the dark. Then, the tube was centrifuged in 1200*g* centrifugal force for 10 minutes at 4°C. The supernatant was decanted. The cells were washed for 3 times and diluted into 400~ 600 *μ*L with PBS. At least 10,000 cells for each sample were obtained and analyzed with Cell Quest 3.0 software. Negative control was set up for all experimental groups to eliminate nonspecific fluorescence. The expression of adhesive molecules (AM) is determined by the fluorescence intensity. Secondly, the changes in expression of adhesion molecules were confirmed by Western blotting. The Western blot analysis was performed according to previously established methods [22].

### 2.10. Measurement of the Expression of HIF-1*α* and NF-*κ*B in BMMSCs

The total protein expression of HIF-1*α* and the total and nuclear level of protein expression of NF-*κ*B in BMMSCs were measured by Western blotting. The Western blot analysis was performed according to previously established methods [22]. Briefly, BMMSCs were collected and lysed in M-PER Mammalian Protein Extraction Reagent (Pierce, Rockford, IL). All samples were normalized according to the protein concentrations and separated in 10% SDS-PAGE gels and then transferred to nitrocellulose filter membranes (Pall Corporation, Washington, NY) using the wet transfer blotting system (Bio-Rad, Hercules, CA). The following antibodies were used for Western blotting: anti-HIF-1*α* (Santa Cruz Biotechnology), anti-NF-*κ*B (Abcam), and anti-GAPDH (Abcam). Goat anti-rabbit secondary antibody was obtained from Santa Cruz Biotechnology. The gray levels of blots were analyzed using ImageJ software.

### 2.11. Statistical Analysis

Data were expressed as means ± SEM, and enumeration data were expressed as percentage. The unpaired Student *t*-test was used to determine the differences between two groups, and the comparison of enumeration data between the two groups was analyzed with *χ*^2^ test. One-way ANOVA (Dunnett's multiple comparison test) was used to determine the differences among the multiple groups. *P* < 0.05 was considered as statistically significant.

## 3. Results

### 3.1. CIHH Treatment Reduced the Incidence Rate of AA

In the CIHH + AA group, 4 out of 20 (20%) rats developed AA. However, 14 out of 20 (70%) rats in the AA group developed AA. So the incidence rate of AA in the CIHH + AA group was significantly lower than that in the AA group (*P* < 0.05).

### 3.2. CIHH Treatment Improved the Reduction of Hematological Parameters in Peripheral Blood of AA Rats

**Figure 1:**
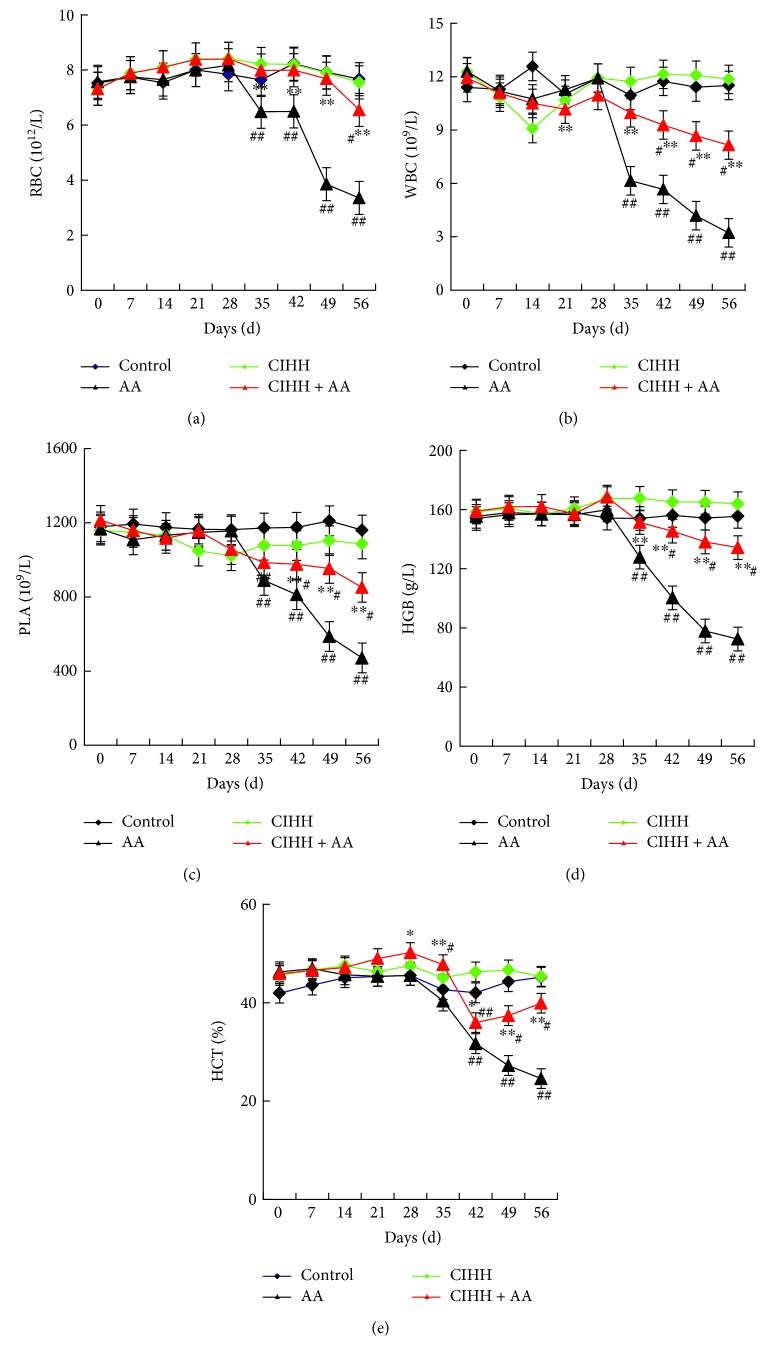
The hematological parameters of peripheral blood in each group. (a–e) The values of RBC, WBC, PLA, HGB, and HCT. ^#^*P* < 0.05 and ^##^*P* < 0.01 versus the control group. ^∗^*P* < 0.05 and ^∗∗^*P* < 0.01 versus the AA group.

AA rats displayed anemia, hemorrhage tendency, and infection symptoms. The body weight of AA rats was decreased significantly. Blood examination results showed that the peripheral blood cells in AA rats were decreased to 1/2 of baseline in 21d AA induction. WBCs were reduced at first; then RBC, PLA, HGB, and HCT were reduced (Figure 1), which matched the characters of AA rats [1]. During following 28d after stopping BU injection, blood hemogram in AA rats was reduced continuously. But the hematological parameters of peripheral blood in CIHH + AA rats could effectively antagonize those deviations of blood compared with those of the AA group (*P* < 0.01). There were no significant differences of hematological parameters of peripheral blood between the control and CIHH group.

### 3.3. CIHH Treatment Improved the Destroyed Bone Marrow Tissue in AA Rats

**Figure 2:**
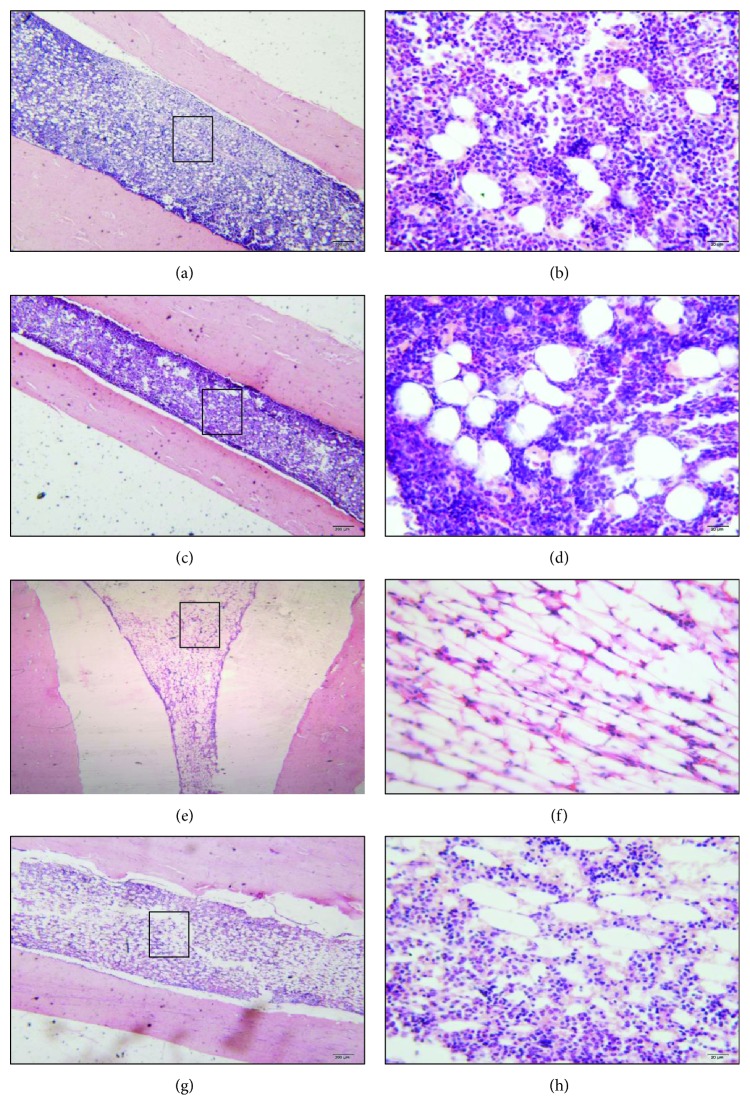
The pathologic morphology of bone marrow tissue in each group. (a, b) Normal bone marrow in the control group; (c, d) normal bone marrow in the CIHH group; (e, f) lessened and damaged bone marrow in the AA group; and (g, h) improved bone marrow in the CHH + AA group. (H-E staining: a, c, e, and g × 40; b, d, f, and h × 400).

There were no significant differences of pathologic morphology in bone marrow tissue between control (Figures 2(a) and 2(b)) and CIHH (Figures 2(c) and 2(d)) rats. While in AA rats, the hematopoietic cells such as megakaryocytes in bone marrow were significantly reduced, the bone marrow hematopoietic scaffold structure was loosened, and the bone marrow reticular fibers were decreased. The number and structure of bone marrow capillary were abnormal, and mesenchymal blood sinus of bone marrow was dilated (Figures 2(e) and 2(f)), while the suppression of bone marrow in CIHH + AA rats were significantly improved compared with AA rats (Figures 2(g) and 2(h)).

### 3.4. CIHH Treatment Increased Both the Hematopoietic Progenitor Cells (HPCs) and Mesenchymal Progenitor Cells (MPCs) in AA Rats

**Figure 3:**
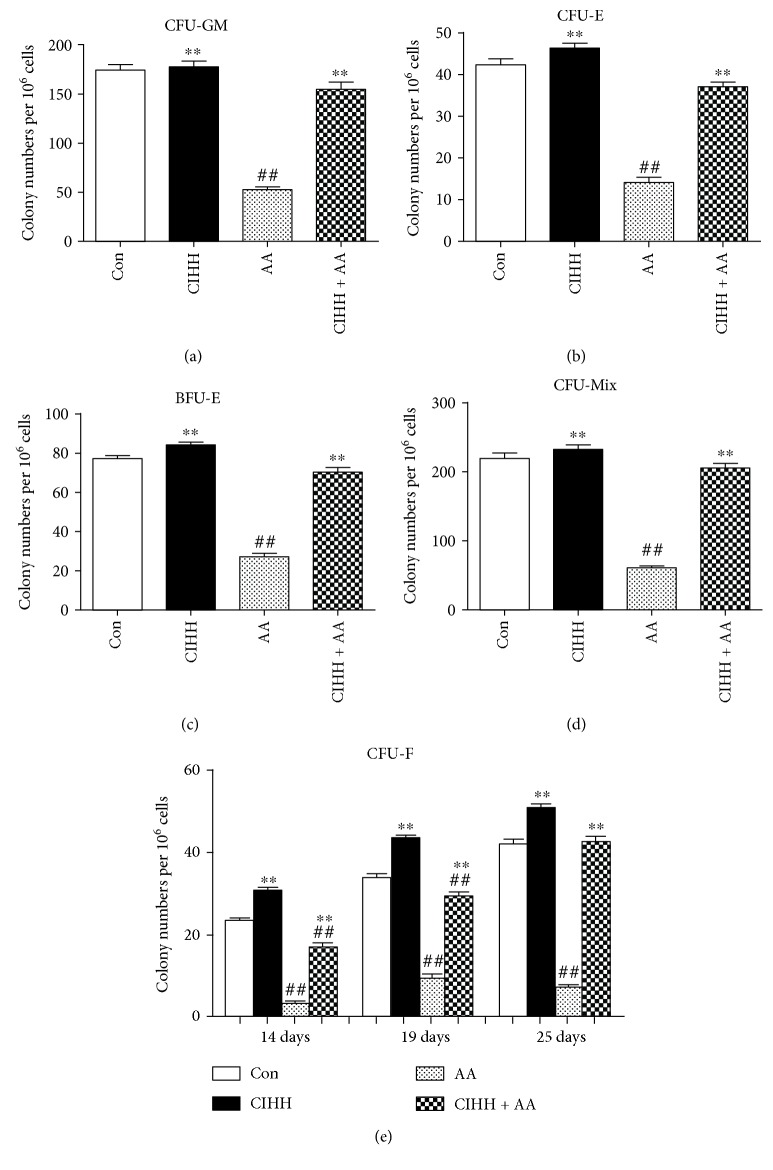
The colony numbers of hematopoietic and mesenchymal progenitor cells in bone marrow of each group. (a–d) Hematopoietic progenitor cells: CFU-GM, CFU-E, BFU-E, and CFU-Mix; (e) mesenchymal progenitor cells: CFU-F. ^##^*P* < 0.01 versus the control group. ^∗∗^*P* < 0.01 versus the AA group.

Progenitor cell assays were performed on the BM samples. As shown in Figures 3(a)–3(d), numbers of HPCs were 174.53 ± 5.75, 77.27 ± 1.55, 42.37 ± 1.43, and 219.33 ± 8.05 in CFU-GM, BFU-E, CFU-E, and CFU-Mix per 10^6^ cells in control rats (*n* = 30), respectively. The numbers of HPCs were 52.77 ± 2.78, 27.20 ± 1.76, 14.10 ± 1.31, and 60.90 ± 2.71 in CFU-GM, BFU-E, CFU-E, and CFU-Mix per 10^6^ cells in AA rats (*n* = 30), respectively (approximately 1/3 of baseline). While in CIHH + AA rats, numbers of HPCs were significantly increased compared with AA rats (*P* < 0.01), which were 154.93 ± 7.56, 70.37 ± 2.35, 37.10 ± 1.14, and 205.63 ± 6.85 in CFU-GM, BFUE, CFU-E, and CFU-Mix per 10^6^ cells, respectively (*n* = 30).

We next examined the colony numbers of MPCs at 14d, 19d, and 25d. A dramatic decrease in MPCs frequency was observed in AA rats (approximately 1/4–1/6 of baseline) (Figure 3(e)). Numbers of MPCs were 3.09 ± 0.68, 9.45 ± 0.90, and 7.09 ± 0.64 at 14d, 19d, and 25d CFU-Fs per 10^6^ cells in AA rats (*n* = 30), respectively. While in CIHH + AA rats, numbers of MPCs were 16.82 ± 0.94, 29.55 ± 1.07, and 42.36 ± 1.57 at 14d, 19d, and 25d CFU-Fs per 10^6^ cells (*n* = 30), respectively, which significantly increased compared with AA rats (*P* < 0.01).

### 3.5. Surface Markers of BMMSCs

**Figure 4:**
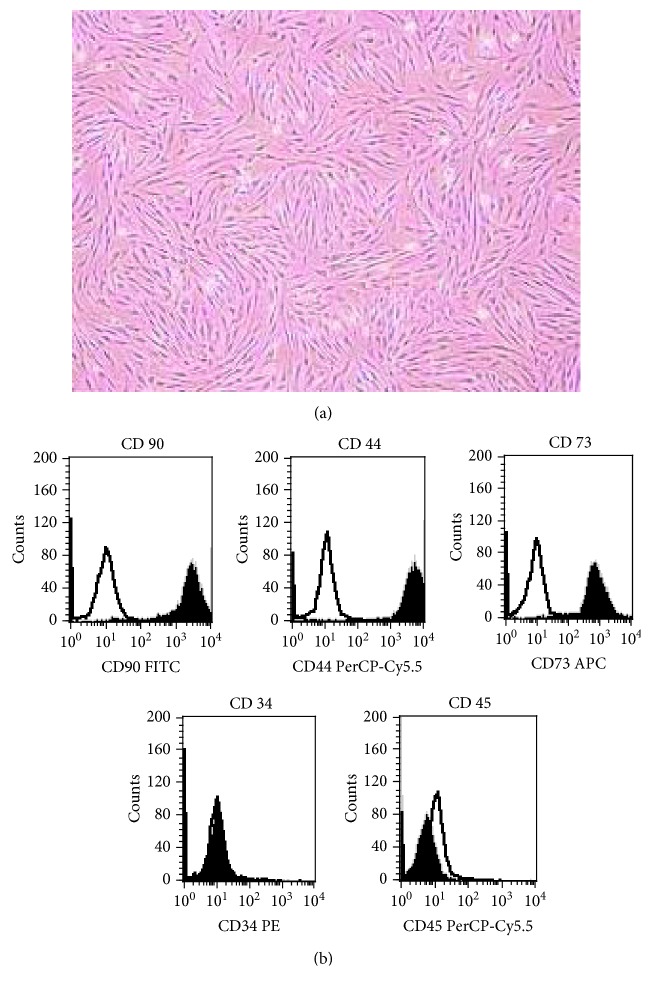
Morphology and surface markers of BMMSCs. (a) Morphology of BMMSCs, observed by phase contrast microscopy. (b) Flow cytometric analysis of the surface markers (CD90, CD44, CD73, CD34, and CD45). Fluorescence intensity histograms with specific antibodies for membrane antigens (black line) and irrelevant isotypic-matched Ab as negative control (black area).

Flow cytometric analysis was performed to assay the surface markers of BMMSCs. The BMMSCs were closely resembled in a homogeneous layer in each group (Figure 4(a)). These cells expressed CD90 (97.3 ± 3.5%), CD44 (98.5 ± 0.6%), and CD73 (96.5 ± 2.5%), while there was an absence of CD34 (2.8 ± 1.7%) and CD45 (1.8 ± 0.5%) (Figure 4(b)).

### 3.6. CIHH Treatment Increased the Expression of VLA-4, VCAM-1, and ICAM-1 and Decreased the Expression of CD162 and CD164 in BMMSCs of AA Rats

**Figure 5:**
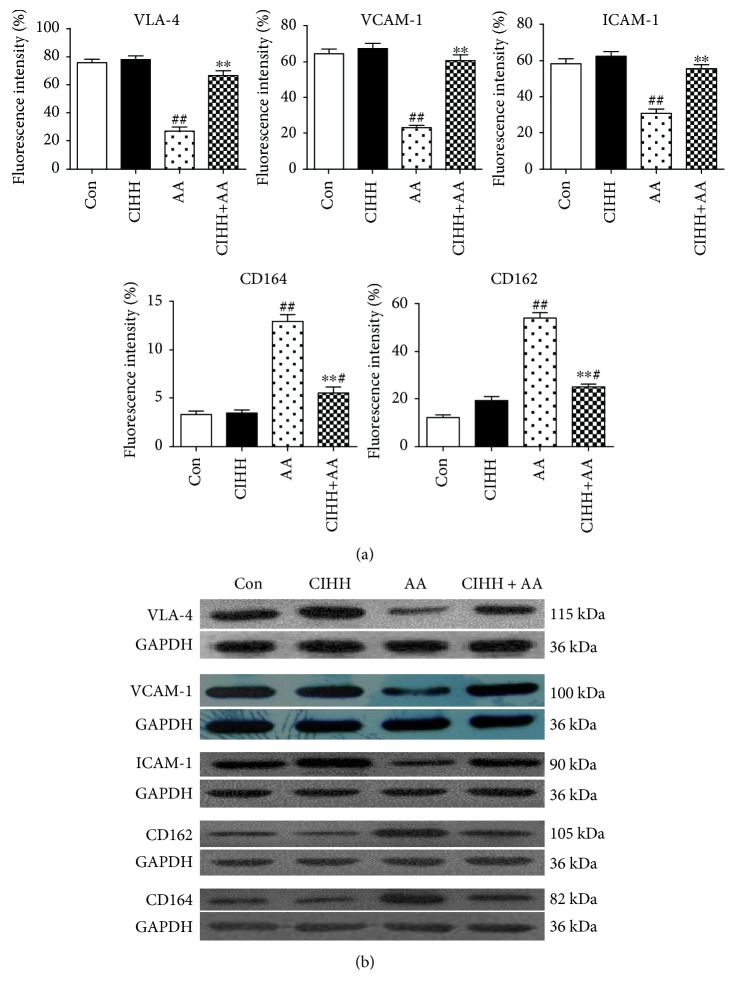
The protein expression of VLA-4, VCAM-1, ICAM-1, CD162, and CD164 in BMMSCs of each group. (a) Fluorescent intensity of VLA-4, VCAM-1, ICAM-1, CD162, and CD164 in BMMSCs by flow cytometry. (b) Representative total protein expression of VLA-4, VCAM-1, ICAM-1, CD162, and CD164 in BMMSCs by Western blotting. ^#^*P* < 0.05 and ^##^*P* < 0.01 versus the control group. ^∗∗^*P* < 0.01 versus the AA group.

Compared with the control group, the protein expression of VLA-4, VCAM-1, and ICAM-1 in BMMSCs of AA rats was significantly decreased (*P* < 0.01), but the expression of CD162 and CD164 was significantly increased in AA rats (*P* < 0.01) while CIHH treatment increased the expression of VLA-4, VCAM-1, and ICAM-1 and decreased the expression of CD162 and CD164 (*P* < 0.01, Figure 5).

### 3.7. CIHH Treatment Decreased the Expression of HIF-1*α* and NF-*κ*B in BMMSCs

**Figure 6:**
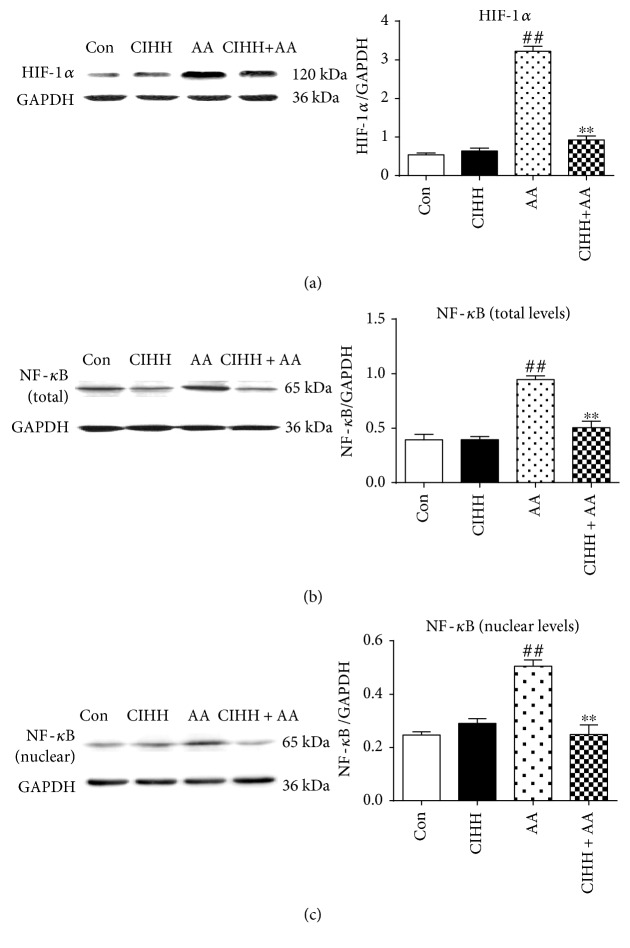
The protein expression of HIF-1*α* and NF-*κ*B in BMMSCs. (a) Representative and quantitative analysis of expression of HIF-1*α* in total levels. (b) Representative and quantitative analysis of expression of NF-*κ*B in total levels. (c) Representative and quantitative analysis of expression of NF-*κ*B in nuclear levels. ^##^*P* < 0.01 versus the control group. ^∗∗^*P* < 0.01 versus the AA group.

Compared with the control group, the protein expression of HIF-1*α* and NF-*κ*B in BMMSCs in AA rats was significantly increased (*P* < 0.01). However, after CIHH treatment, the protein expression of HIF-1*α* and NF-*κ*B was significantly decreased in BMMSCs in CIHH + AA rats (*P* < 0.01, Figure 6).

## 4. Discussion

In the present study, CIHH treatment reduced the incidence rate of AA, improved the abnormality of hematological parameters in peripheral blood of AA rats, and restored the destroyed bone marrow tissue in AA rats. CIHH treatment also increased the colony numbers of both hematopoietic progenitor cells (HPCs) and mesenchymal progenitor cells (MPCs) in AA rats. CD90, CD44, and CD73 were expressed on BMMSCs, while there was an absence of CD34 and CD45. CIHH treatment increased the expression of VLA-4, VCAM-1, and ICAM-1 in BMMSCs of AA rats but decreased the expression of CD162 and CD164. CIHH treatment decreased the expression of HIF-1*α* and NF-*κ*B in BMMSCs.

Although several studies suggested that the HSC damage, immunity disorder, and defects on hematopoietic microenvironment might play roles in the development of bone marrow failure in AA, the exact mechanism for AA is still not clear. Traditional treatment of AA focuses on the repair of damaged HSCs and regulation of immune function. The advanced research found that improving HIM of bone marrow in AA patients will promote the curative effect for AA with a doubled result. Research has shown that there is the damage of HSCs and HIM [32] in AA rat model induced by BU.

The result in our study showed that CIHH could effectively antagonize the decreasing of bone marrow CFU-F, recover the mature fibroblast-like stromal cell layer that formed in bone marrow, and support the hematopoiesis of bone marrow in AA rats. The in vitro study revealed that persistent hypoxia stimulates CFU-F proliferation [33], but we do not know the effect of CIHH on CFU-F. Combining the studies of ours and others, we guess that the enhancement of CIHH on CFU-F (1) stimulates T lymphocyte of bone marrow to produce positive hematopoietic regulator, such as IL-3 and stem cell factor and to promote hematopoietic cells growth and proliferation [34] and (2) improves autocrine and paracrine of BMMSCs. The fibroblasts in the colonies are the main component of stromal cells in bone marrow, which can reflect the function of BMMSCs to a certain degree [35].

There is a great number of adhesion ligands on the surface of BMMSCs, which can combine to adhesion receptors on the surface of hematopoietic stem/progenitor cells. So BMMSCs are often described as the soil of HSCs and play a key role in hematopoiesis through stimulating the proliferation and differentiation of hematopoietic stem/progenitor cells [36] or through the extracellular medium [7]. Therefore, the abnormality of adhesion ligand expression in BMMSCs will affect the growth of hematopoietic stem/progenitor cells in the bone marrow microenvironment. It is reported that the expression of BMMSC adhesion molecules in AA patients was low [37], the level of SCF was decreased, and the BMMSCs grew slowly and were easy to differentiate into adipocytes. These adhesion molecules constitute a complex supporting hematopoiesis network, which is the molecular basis of the support of hematopoiesis by stromal cells [38]. On the other hand, hypoxia can regulate the bone marrow hematopoietic microenvironment [5], improve the capacity of bone marrow, and increase the expression of certain adhesion molecules in bone marrow cells [39].

In this study, we focus on the adhesion molecules VLA-4, VCAM-1, ICAM-1, CD162, and CD164. VCAM-l and VLA-4 are involved in the adhesion process between bone marrow hematopoietic stem cells and mesenchymal stem cells. VLA-4 plays a role mainly in adhesion between cell and extracellular matrix and is related to cell migration and differentiation [40]. Studies have shown that the adhesion of bone marrow hematopoietic cells and bone marrow stromal cells/extracellular matrix mediated by VLA-4 is the basis of the bone marrow cell proliferation. ICAM-1 is widely distributed in hematopoietic and nonhematopoietic cells and can regulate the adhesion between hematopoietic progenitor cells and bone marrow stromal cell/extracellular matrix layer. The expression of VCAM-1 can be increased by the stimulation of IL-l *β*, IL-4, or TNF-*α* and is a key factor in hematopoietic cell proliferation and differentiation. Studies showed that the direct expression of ICAM-1 is closely correlated with the clinical efficacy on AA treatment. CD162 is expressed in hematopoietic progenitor cells, lymphocytes, and granulocytes and is the only receptor of CD62P (P-selectin) [41]. CD162 mRNA can be expressed in mature CD34^+^ cells, and the capacity of CD162 in combination with P-selectin is gradually decreased along with CD34^+^ cell differentiation and matures [42]. There are two possible mechanisms for the inhibition of CD162 on the proliferation of CD34^+^ cells. One is that the adhesion between CD162 and CD34^+^ cells mediates the apoptosis of CD34^+^ hematopoietic stem/progenitor cells. The other is that the differentiation of CD34^+^ hematopoietic stem/progenitor cells into cell proliferation cycle is inhibited [43]. But the exact mechanism for CD162 action is unclear yet. CD164, expressed in hematopoietic stem/progenitor cells, lymphocytes, macrophages/monocytes, and epithelial cells, plays a role in the adhesion and proliferation in hematopoietic stem/progenitor cells and bone marrow stromal cells [44]. CD164 promotes the adhesion between CD34^+^ cells and bone marrow stromal cells and inhibits the proliferation of the hematopoietic stem/progenitor cells at the same time [45]. So CD164 is a negative regulatory factor on the proliferation of hematopoietic progenitor cells [46].

The adhesion dysfunction may reduce the adhesion of hematopoietic cells and mobilize excessive pluripotent stem cells from the adhesion area into the blood circulation which may be destroyed, resulting in bone marrow hematopoietic failure eventually. However, CIHH treatment can effectively antagonize the adhesion dysfunction in AA rats and recover the hematopoietic microenvironment. There are two possible mechanisms for CIHH anti-AA through adhesion molecule action: (1) CIHH can improve the adhesiveness by regulating the expression of BMMSC adhesion molecules in AA rats and promoting BMMSCs to secrete more hematopoietic factors. And CIHH also can enhance the sensitivity of BMMSCs to hematopoietic factors and improve the homing of hematopoietic cells back to the marrow, which promotes the homing of different stages of hematopoietic cell into the hematopoietic specific area in bone marrow. (2) CIHH treatment can make the pluripotent stem cells continuously into niche to be saved up through regulating the BMMSC adhesion function.

Finally, we investigated the effect of CIHH on protein expression of HIF-1*α* and NF-*κ*B in BMMSCs of AA rats. NF-*κ*B and HIF-1*α* are two important signal transduction molecules involved in hypoxia, inflammation reactions, and stress under physiological and pathological conditions. But it is not clear if the role of NF-*κ*B and HIF-1*α* in AA. HIF-1*α* is a nuclear transcription factor that plays a role in hypoxic condition. HIF-1*α* regulates the expression of angiogenesis-related genes, apoptosis-related gene, and EPO [47]. It is known that the normal bone marrow cavity itself is in hypobaric hypoxia condition. Qian et al. [48] found that HIF-1*α* could activate caspase-3 and promote apoptosis of bone marrow stromal cells in hypoxia and reoxygenation condition. If the hypoxia microenvironment in bone marrow is aggravated in AA, caspase-3 can be activated and apoptosis of bone marrow cells can be induced. NF-*κ*B is a kind of eukaryotic transcription factor and participates in the expression of genes regulating immune, inflammation, apoptosis, and cell proliferation process [49]. NF-*κ*B signal pathway can be activated by various stress stimulators such as cytokines, bacteria, virus, ultraviolet radiation, and free radical. The activated NF-*κ*B protein can enhance not only the transcription level of cytokines but also acute stress reactive protein gene [50]. Walmsley et al. found that the proximal HIF-1*α* gene promoter site contained the activated NF-*κ*B-binding site [51]. So the hypoxia could raise the transcription of HIF-1*α* through NF-*κ*B pathway [52]. The overexpression of HIF-1*α* can promote NF-*κ*B activity and increase the reaction of stress [53]. The latest research showed that NF-*κ*B protein plays a key role in the development of hematopoietic cells [54]. Both signaling pathways of NF-*κ*B and HIF-1*α* in AA rats could promote the expression for each other through crosstalking, resulting in excess stress of bone marrow cells in AA rats. CIHH has an effective effect against the increase of stress reaction, which will inhibit the overexpression of NF-*κ*B and HIF-1*α* ultimately.

In conclusion, this study demonstrated for the first time that CIHH has an antiaplastic anemia effect, which might be related to the improvement on adhesiveness and stress of mesenchymal stem cells.
